# CLINICAL PRESENTATION OF INTERSTITIAL LUNG DISEASE AS AN INITIAL MANIFESTATION OF ANTISYNTHETASE SYNDROME

**DOI:** 10.1093/geroni/igae098.2912

**Published:** 2024-12-31

**Authors:** Pamela Salcido De Pablo, Carolina Escorcia Encarnación, Alfredo Olvera

**Affiliations:** University Anahuac Queretaro, Queretaro, Queretaro de Arteaga, Mexico; Universidad Anahuac, Querétaro, Queretaro de Arteaga, Mexico; Universidad Anáhuac Querétaro, Educación, Distrito Federal, Mexico

## Abstract

Antisynthetase syndrome (ASS) is an autoimmune disease, categorized as a type of idiopathic inflammatory myopathy, where interstitial lung disease is the most common manifestation, presenting in up to 80% of cases. It is related to antibodies and aminoacyl-transfer RNA (tRNA) and the most common one, anti-Jo. It has an incidence of 0.56 per 100,000 inhabitants, commonly in adults with an average age of 45 to 70 years old. A 72-year-old female, with a medical history of dyslipidemia, depression, and gonarthrosis, presents with dyspnea, hypoxemia, wheezings, asthenia, arthralgias, and myalgias. A chest radiograph showed abnormal parenchyma, with bilateral opacities in peripheral and subpleural zones, diagnosis was compatible with pneumonitis, treated with quinolones, steroids, and short-acting bronchodilators. With partial recovery, TC scan showed patchy ground-glass-like pattern, bronchi ecstasies, and honeycomb zones, spirometry showed restrictive pattern and rheumatology tests reports were negative for antibody anti-Jo-1 and positive in antinuclear antibodies (ANAs). After several differential diagnoses and studies; a diagnosis of antisynthetase syndrome was reached. Although its low prevalence in the general population, the importance of knowing about antisynthetase syndrome lies in understanding the pathology and encouraging research on it, thus achieving a good diagnosis and adequate interdisciplinary management that these patients should require in a syndrome that still exists. For this reason, this case report intends to mention some of the most used tools currently in use, which helped to diagnose this interesting and uncommon case.

